# A Card

**Published:** 1875-04

**Authors:** 


*A Card.*

We desire to call the attention of the Faculty to the following analyses of Cincho-Quinine, from some of the most eminent chemists. BILLINGS, CLAP & CO., *Boston.*

---

CHEMICAL LABORATORY OF UNIVERSITY OF PENNSYLVANIA,
*West Philadelphia*, January 29, 1875.

*Messrs. Billings, Clapp & Co.:*

GENTLEMEN,—I have received by express a package marked, "Sealed by S. P. Sharples, January 22, 1875," and containing a bottle of Cincho-Quinine with the label of James R. Nichols & Co., Chemists, Boston, which I have tested, and find it to contain quinine, quinidine, cinchonine, and cinchonidine.

Yours, respectfully, F. A. GENTH,
*Professor of Chemistry and Mineralogy.*

---

LABORATORY OF THE UNIVERSITY OF CHICAGO,
*Chicago*, February 1, 1875.

I hereby certify that I have made a chemical examination of the contents of a bottle of Cincho-Quinine, and by direction I made a qualitative examination for quinine, quinidine and cinchonine, and hereby certify that I found these alkalies in Cincho-Quinine.

C. GILBERT WHEELER, *Professor of Chemistry.*

---

THE chemical analyses of Cincho-Quinine, given above, certainly prove that it is a valuable preparation, and suggest that, in many cases, it is a superior tonic to quinine. Therefore, it should be kept in every drug store.

